# Cardiac Sarcoidosis: A Case Report on the Role of Cardiac Computed Tomography (CT) in the Evaluation of Advanced Atrioventricular Block

**DOI:** 10.7759/cureus.39479

**Published:** 2023-05-25

**Authors:** Christopher Marsalisi, John M Sousou, Hui Jun Guo, Tanya Deol, Loruanma Lam, Wakil Irfan, Spencer G Streit

**Affiliations:** 1 Internal Medicine, University of Florida College of Medicine- Jacksonville, Jacksonville, USA; 2 Medical Student, Lake Erie College of Osteopathic Medicine, Jacksonville, USA; 3 Cardiology, Mayo Clinic, Jacksonville, USA

**Keywords:** ct cardiac, positron emission tomography computed tomography, infiltrative cardiomyopathy, complete heart block, cardiac sarcoidosis, systemic sarcoidosis

## Abstract

Cardiac sarcoidosis is a rare autoimmune condition that is characterized by the presence of non-caseating granulomas in the cardiac tissue. We present the case of a 31-year-old male with no significant past medical history who presented with palpitations and lightheadedness during exertion for two to three months and was found to have complete heart block on his 12-lead electrocardiogram. A cardiac CT was obtained to rule out an ischemic event, but it indicated findings suggestive of pulmonary sarcoidosis. The CT findings helped tremendously with narrowing down the differential diagnosis and providing efficient diagnostic and therapeutic management.

## Introduction

Sarcoidosis is a multisystemic condition of unknown etiology that is characterized by the presence of inflammatory noncaseating granulomas. The incidence of sarcoidosis varies across the world depending on race and ethnicity, with the highest being seen among northern Europeans, African Americans, and Canadians [1]. Sarcoidosis is unique in that it has the potential to affect any organ system in the body; however, the most common sites of involvement are the lungs, mediastinal lymphatic nodes, hepatic parenchyma, the spleen, and the integumentary system. One of the least common sites affected by sarcoidosis is the heart, with only about 5% of patients with confirmed sarcoidosis having involvement in this tissue [2].

The characteristic granulomas associated with cardiac sarcoidosis deposit in several areas of the heart, with the myocardium of the left ventricular free wall being the most common [3]. In these cases, the extent of myocardial involvement and the presence of left ventricular (LV) dysfunction can predict the overall prognosis for the given patient. Common symptomatic features of cardiac sarcoidosis are oftentimes nonspecific; however, they typically include chest pain, heart failure symptoms, syncope, fatigue, and palpitations. Objective clinical findings may be present in electrocardiographic studies (EKG), with common findings including varying degrees of conduction block, QRS prolongation, pathological Q waves, and arrhythmias such as ventricular tachycardia [2].

In the present case, we discuss the diagnostic workup and management of a patient with a complete heart block who underwent a permanent pacemaker (PPM) implantation. He subsequently developed sustained ventricular arrhythmia which required his pacemaker to be upgraded to an implantable cardioverter-defibrillator. During his hospitalization, the patient was diagnosed with cardiac sarcoidosis and was discharged in stable condition, with follow-up visits demonstrating an appropriate response to immunosuppressive therapy.

## Case presentation

A 31-year-old male with no significant past medical history presented to the emergency room (ER) from his primary care provider’s office due to concern for bradyarrhythmia. The patient initially presented to his primary care physician’s office after experiencing 2-3 months of intermittent palpitations during exertional activities, along with lightheadedness, a dry cough, shortness of breath, wheezing, and several near-syncopal episodes.

**Figure 1:**
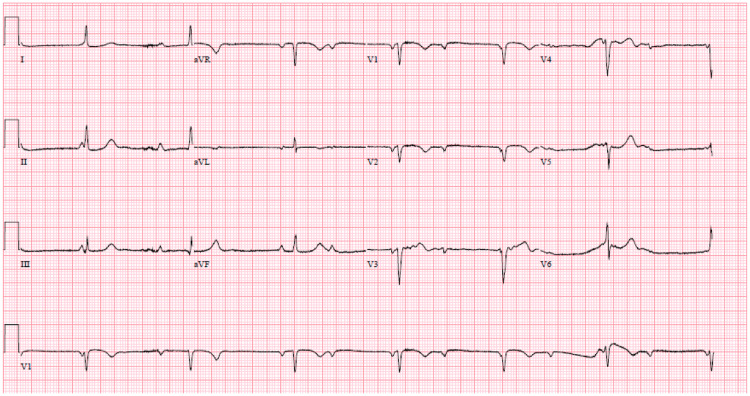
12-lead EKG demonstrating normal sinus rhythm with complete atrioventricular block and junctional escape rhythm at 42 bpm. EKG: electrocardiogram; BPM: beats per minute

In the ER, the patient was asymptomatic and hemodynamically stable, with an unremarkable initial laboratory analysis. The EKG indicated a complete heart block with a junctional escape rate of 53 beats per minute (Figure 1). The patient was admitted to the cardiac care unit (CCU) for further workup and management.

The patient’s initial chest X-ray demonstrated bilateral patchy interstitial opacities, with a follow-up cardiac CT scan indicating biventricular and right atrial enlargement and diffuse bilateral pulmonary micronodules with mediastinal and hilar lymphadenopathy, highly suspicious for sarcoidosis. The transthoracic echocardiogram (TTE) redemonstrated the aforementioned cardiac chamber abnormalities and estimated the left ventricular ejection fraction (LVEF) at 55-60%. In the setting of conduction abnormalities with imaging findings concerning granulomatous disease, a preliminary diagnosis of cardiac sarcoidosis was considered.

**Figure 2:**
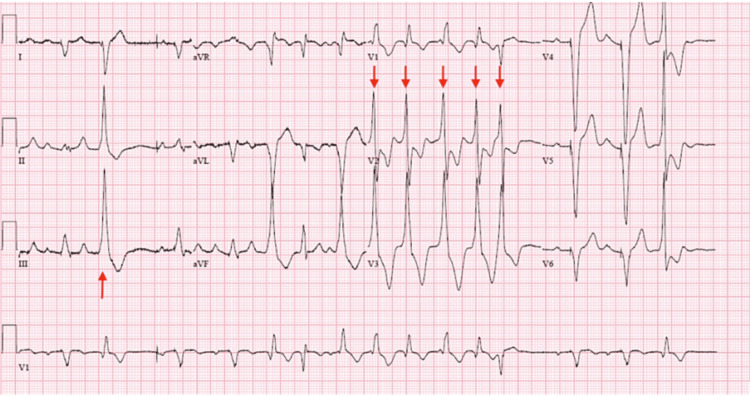
NSVT and PVCs with right bundle branch morphology and inferior axis likely originating from the left ventricular basal area. NSVT: non-sustained ventricular tachycardia; PVCs: premature ventricular complexes

The patient was monitored in the CCU while the initial workup was ongoing. However, he became symptomatic and underwent permanent pacemaker implantation two days after admission. He subsequently developed premature ventricular complexes (PVCs) and runs of non-sustained ventricular tachycardia (NSVT) (Figure 2). These arrhythmias showed no improvement after the initiation of sotalol, so his device was upgraded to an implantable cardioverter-defibrillator (ICD), and his beta-blocker dose was uptitrated. During the rest of his hospitalization, he did not experience any additional episodes of tachyarrhythmias.

In the interim of the treatment course, an endobronchial biopsy of one of the mediastinal lymph nodes was performed, which demonstrated non-caseating chronic granulomatous inflammation. The patient was discharged in stable condition and was instructed to follow up with cardiology and rheumatology for further workup of systemic sarcoidosis.

**Figure 3:**
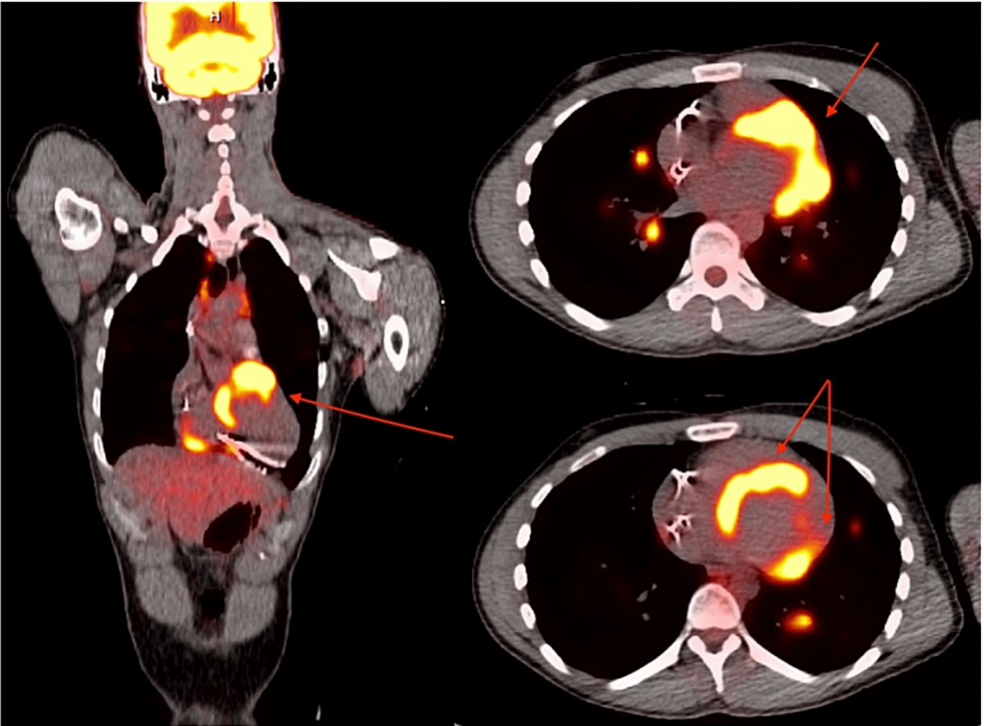
Cardiac PET scan indicating FDG uptake in the septal basal lateral, inferior, and anterior walls with additional uptake in the RV basal wall. PET: positron emission tomography; FDG: fluorodeoxyglucose; RV: right ventricle

A positron emission tomography (PET) scan was ordered, which showed diffuse fluorodeoxyglucose (FDG) uptake in the left ventricle (LV) septal basal lateral, inferior, and anterior walls, with additional uptake in the right ventricle (RV) basal wall (Figure 3). The scan also indicated multiple pulmonary nodules as well as supraclavicular, mediastinal, perihilar, and inguinal fluorodeoxyglucose avid lymph nodes. Due to a very high suspicion of uncontrolled sarcoidosis, the patient was started on maintenance therapy with methotrexate. After starting therapy, the patient showed significant improvement in his respiratory symptoms, which were his initial complaint when presenting to his primary care physician (PCP) prior to hospital admission.

## Discussion

Cardiac sarcoidosis is a relatively rare condition, with only about five percent of those diagnosed with sarcoidosis having clinically significant cardiac involvement. Furthermore, many patients with pulmonary and systemic sarcoidosis have clinically silent cardiac involvement. Symptomatic patients with cardiac sarcoidosis may present with chest pain, shortness of breath, palpitations, arrhythmia, or atrioventricular block [4].

TTE usually demonstrates non-specific findings such as interventricular thinning, left ventricular hypertrophy, systolic or diastolic dysfunction, septal thinning, ventricular aneurysms, or isolated wall motion abnormalities [3]. Due to the non-specific nature of clinical symptoms, EKG findings, and echocardiography results, cardiac sarcoidosis may sometimes be misdiagnosed. Some similar and more common conditions that can mimic the clinical presentation of cardiac sarcoidosis include dilated cardiomyopathy, heart failure, and hypertrophic cardiomyopathy. Although TTE may aid in the diagnosis of cardiac sarcoidosis, cardiac magnetic resonance imaging (MRI) remains the most effective diagnostic imaging tool. Given its high sensitivity, estimated to be about 93%, cardiac MRI allows for a multidimensional evaluation of myocardial damage through the use of late gadolinium enhancement (LGE) [2]. This is a relatively newer diagnostic technique utilized for the detection of minuscule degrees of fibrosis and scar tissue.

In the presented case, cardiac CT demonstrated pulmonary nodules and lymphadenopathy, which played a significant role in raising the team's suspicion that cardiac sarcoidosis was the etiology of advanced atrioventricular (AV) block. This speculation, however, should not be interpreted as overestimating the utility of cardiac CT in patients with cardiac sarcoidosis. The cardiac CT can only aid in exploring differentials, and the definitive diagnosis of cardiac sarcoidosis is made by appropriate clinical presentation (atrioventricular block (AVB), ventricular tachycardia (VT), and unexplained systolic heart failure) and positive advanced cardiac imaging with fluorodeoxyglucose-positron emission tomography (FDG-PET) or cardiac magnetic resonance (CMR) imaging.

The mainstay of treatment for cardiac sarcoidosis is the use of corticosteroids to decrease inflammation and prevent fibrosis of the myocardium [5]. Other effective treatments that may be used alongside corticosteroids include immunosuppressive agents and guideline-directed medical therapy for heart failure if there is significant left ventricular dysfunction. Patients who develop advanced heart block typically require the implantation of a pacemaker or ICD if ventricular arrhythmias are appreciated.

Although in some patients advanced AV block may recover with steroid therapy, in other patients it persists. In fact, the CT findings raised our concern for sarcoidosis so much that we proceeded directly to a permanent pacemaker and thus avoided an additional invasive procedure such as a transvenous pacemaker. The patient developed a ventricular arrhythmia, and this prompted us to upgrade the pacemaker to an ICD for primary prevention of sudden death. To suppress frequent PVCs and episodes of NSVT and avoid ICD shocks, we uptitrated the dose of sotalol.

## Conclusions

This case highlights the role of cardiac CT in the diagnostic workup of young patients who present with exertional symptoms such as palpitation and lightheadedness. Cardiac CT has the potential to 1) rule out the ischemic etiology of the symptoms and 2) reveal cardiac and extracardiac findings that are related to the arrhythmic etiology of the symptoms. In relation to the presented case, the presence of pulmonary nodules and lymphadenopathy on CT in the context of advanced AV nodal block narrowed the team’s differentials and raised suspicion for sarcoidosis, assisted in a timely diagnosis, and helped guide treatment.
